# CACNA2D3 Enhances the Chemosensitivity of Esophageal Squamous Cell Carcinoma to Cisplatin via Inducing Ca^2+^-Mediated Apoptosis and Suppressing PI3K/Akt Pathways

**DOI:** 10.3389/fonc.2019.00185

**Published:** 2019-04-02

**Authors:** Changjun Nie, Xiaohui Qin, Xiaoyan Li, Baoqing Tian, Ying Zhao, Yuan Jin, Yadan Li, Qiang Wang, Dingyuan Zeng, An Hong, Xiaojia Chen

**Affiliations:** ^1^Department of Cell Biology, Institute of Biomedicine, Jinan University, Guangzhou, China; ^2^National Engineering Research Center of Genetic Medicine, Guangzhou, China; ^3^Guangdong Provincial Key Laboratory of Bioengineering Medicine, Guangzhou, China; ^4^Department of Medical Genetics, Liuzhou Maternal and Children Healthcare Hospital, Liuzhou, China

**Keywords:** CACNA2D3, chemosensitivity, ESCC, voltage-gated calcium channel, LY294002

## Abstract

Resistance to platinum-based combination chemotherapy is the main cause of poor prognosis in patients with advanced esophageal squamous cell carcinoma (ESCC). Previously, we showed that CACNA2D3 (voltage-dependent subunit alpha 2 delta 3 of a calcium channel complex) was significantly downregulated and functioned as a tumor suppressor in ESCC, but its role in the chemosensitivity of ESCC to cisplatin remained unknown. Here, we found that the expression of CACNA2D3 was significantly associated with poor platinum response in ESCC patients from the Gene Expression Omnibus database. Overexpression of CACNA2D3 increased sensitivity to cisplatin in ESCC *in vitro*, whereas knockdown of CACNA2D3 increased cisplatin resistance. CACNA2D3 promoted cisplatin-induced apoptosis by modulating intracellular Ca^2+^ stores. *In vivo* experiments further showed that overexpression of CACNA2D3 enhanced cisplatin anti-tumor effects in a xenograft mouse model. CACNA2D3 overexpression also resulted in the attenuation of PI3K and Akt phosphorylation. Treatment with the PI3K/Akt inhibitor LY294002 restored the chemosensitivity of CACAN2D3-knockdown cells to cisplatin. In conclusion, the results of the current study indicate that CACAN2D3 enhances the chemosensitivity of ESCC to cisplatin via inducing Ca^2+^-mediated apoptosis and suppressing PI3K/Akt pathways. Therefore, regulating the expression of CACNA2D3 is a potential new strategy to increase the efficacy of cisplatin in ESCC patients.

## Introduction

Esophageal cancer (EC) is a fatal digestive tract malignancy (1). EC is composed of two major histologic subtypes: adenocarcinoma and squamous cell carcinoma. Esophageal squamous cell carcinoma (ESCC) is more common in Southeast and Central Asia (2, 3). China is a high incidence area for ESCC, especially in Linzhou and Cixian of North China (4). Esophagectomy is the usual method for the treatment of early esophageal cancer. However, most ESCC patients are diagnosed at an advanced stage when surgery is no longer effective. Recently, the use of comprehensive perioperative therapies has dramatically improved the therapeutic efficacy of ESCC, particularly with respect to long-term survival (5–7). A cisplatin-based regimen is widely used as the first-line treatment in advanced ESCC (8–10). However, cisplatin chemotherapy is often limited by natural and acquired resistance. Consequently, it is critical to identify potential resistance mechanisms in order to restore tumor cell sensitivity to cisplatin.

The human *CACNA2D3* gene is located on the short arm of chromosome 3 at position 3p21.1, a common region of allelic deletion, and has been found to possess a potential tumor suppressor function in multiple tumor types, including gastric cancer (11–13), nasopharyngeal cancer (14), breast cancer (15), renal cell cancer neuroblastoma (16), lung cancer (17), and glioma (18). The promoter of CACNA2D3 was shown to be highly methylated in gastric cancer, and this was associated with a low survival rate (12). Similarly, suppression of CACNA2D3 by methylation was found to promote the metastatic phenotype of breast cancer (15). Another study showed that CACNA2D3 could increase intracellular Ca^2+^ levels and promote apoptosis in nasopharyngeal cancer and glioma, causing changes in the network of tumor-suppressive properties and inducing upregulation of Nemo-like kinase (NLK) through the non-canonical Wnt/Ca^2+^ signaling pathway (14, 18). In neuroblastomas with poor prognosis, the expression of CACNA2D3 is often downregulated (19, 20). Our previous study also identified CACNA2D3 as a tumor suppressor gene, and methylation of its promoter and allele deletion could inhibit its expression in ESCC (21). Recently, CACNA2D3 was implicated in the development of chemoresistance. The downregulation of CACNA2D3 was detected in five cytarabine-resistant leukemic cell lines compared with parental cells (22). However, the underlying mechanism by which CACNA2D3 might function in chemosensitivity has not been identified.

In this study, we aimed to investigate the function of CACNA2D3 in cisplatin-based chemotherapy of ESCC and discover its underlying mechanisms. We found that the expression of CACNA2D3 was significantly associated with poor platinum response in ESCC patients. Overexpression of CACNA2D3 significantly sensitized ESCC cell lines to cisplatin, while CACNA2D3 knockdown induced cellular resistance to cisplatin. Further research showed that CACNA2D3 overexpression enhanced cisplatin-induced apoptosis by modulating intracellular Ca^2+^. Moreover, CACNA2D3 overexpression resulted in the attenuation of PI3K and Akt phosphorylation. LY294002 is a commonly used PI3K/AKT pathway inhibitor, and treatment with LY294002 could restore the chemosensitivity of CACAN2D3-knockdown cells to cisplatin.

## Materials and Methods

### Cell Lines and Reagents

Six ESCC cell lines (KYSE30, KYSE140, KYSE180, KYSE410, KYSE510, and KYSE520) were purchased from DSMZ, the German Resource Centre for Biological Material (23). The short tandem repeat (STR) analysis technique was used to periodically identify all cell lines. Cell lines were cultured in RPMI1640 medium (Hyclone, Logan, UT, USA) supplemented with 10% fetal bovine serum and 1 × penicillin/streptomycin (100 units/mL, 100 μg/mL) (Gibco, NY, USA) at 37°C in a humidified incubator (5% CO_2_/95% air). Cisplatin was acquired from Sigma. LY294002 was purchased from Selleck.

### Plasmid Constructs and Stable Transfection

CACNA2D3 cDNA was amplified from normal human esophageal epithelial cells. The eukaryotic expression vector pcDNA3.1 (+) (Invitrogen, Carlsbad, CA, USA) was used for cloning the human CACNA2D3 gene. Then pcDNA3.1-CACNA2D3 was transfected into the ESCC cell line KYSE30 using Lipofectamine™ 3000 (Invitrogen, Carlsbad, CA, USA). The empty vector was used as a negative control. KYSE30 cells stably expressing CACNA2D3 were screened with 500 μg/ml G418.

### RNA Interference

Small interfering RNA (siRNA) (SR310953) targeting CACNA2D3 and scrambled negative control siRNA (SR30004) were purchased from OriGene. After transfection for 48 h, the relative expression of CACNA2D3 was detected by quantitative real-time PCR (qRT-PCR) and western blotting.

### Cell Viability Assay

A Cell Counting Kit-8 (CCK-8) assay (Dojindo, Kumamoto, Japan) was performed to measure cell viability. Cells were seeded at a density of 1 × 10^4^ cells/well in 96-well plates and incubated with serial dilutions of cisplatin for 72 h. The CCK-8 reagent and RPMI-1640 were diluted in a 1:9 ratio and used to replace the original medium. After incubation at 37°C for 2.5 h, absorbance at a wavelength of 450 nm was measured using a microplate reader. Three independent experiments were conducted. Half maximal inhibitory concentration (IC50) was calculated to evaluate cell resistance to cisplatin using GraphPad Prism 5.0.

### Colony Formation Assay

Cells were seeded at a density of 1.5 × 10^3^ cells/well in six-well plates and treated with respective concentrations of cisplatin. After incubation for 10–14 days, the cell colonies were fixed with ethanol for 30 min and then stained with 0.1% crystal violet for 15 min. Colonies (≥50 cells) were counted. All assays were independently performed in triplicate.

### Intracellular Calcium Assay

The fluorescent probe Fluo-3 AM assay (Beyotime, Haimen, China) was used to measure intracellular Ca^2+^ concentrations. Cells were washed twice with phosphate-buffered saline (PBS) and then loaded with Fluo-3 AM (1 μM) for 30 min in the dark at 37°C. When Fluo-3 AM penetrates the cellular membrane, it is hydrolyzed by cellular esterases to Fluo-3. Fluo-3 emits green fluorescence when combined with Ca^2+^. The intracellular Ca^2+^ concentration was measured by flow cytometric analysis.

### Apoptosis Assays

Apoptosis in ESCC cells treated with or without cisplatin were evaluated using an Annexin V-FITC/propidium iodide (PI) kit (Beyotime, Haimen, China). In brief, cells were digested, washed, and centrifuged twice with cold PBS. After fixing in 75% ethanol for 3 h, cells were stained with Annexin V-FITC and PI at room temperature for 30 min. Then, cells were measured by flow cytometry with FL-1 (530 nm) and FL-2 (585 nm) at an excitation wave length of 480 nm. The data were quantified using the FlowJo software.

### Mitochondrial Membrane Potential Array

The mitochondrial membrane potential (ΔΨm) was analyzed using a JC-1 assay kit (Beyotime, Haimen, China). JC-1 is a fluorescent probe for detecting mitochondrial membrane potential; it accumulates in the mitochondrial matrix and forms a red fluorescent polymer under high membrane potential. When the mitochondrial membrane potential is low, JC-1 exists as a monomer and produces green fluorescence. The cells were trypsinized and stained with JC-1 (10 mg/mL) at 37°C for 20 min. After being washed twice in PBS, cells were analyzed by flow cytometry using emission wavelengths of 590 and 525 nm.

### Quantitative Real-Time PCR

Total RNA was extracted from ESCC cells using TRIzol reagent (Invitrogen, Carlsbad, CA, USA). First-strand cDNA was synthesized using the Reverse Transcription System (Promega, Wisconsin, USA), and mRNA expression levels were measured by qRT-PCR using a CFX96 Touch™ Real-Time PCR Detection System with SYBR Green Dye mixes (Applied Biosystems, Foster City, CA, USA). The PCR primers used for q-PCR were as follows. CACNA2D3: forward (5′-AGGGATTCACGGTTATGCCTT-3′), reverse (5′-GCCACACCTAAACCCTTTGTC-3′); β-Actin, forward (5′-GCTTGTCCAAGAGTGCATGGT-3′), reverse (5′-CAGGGCTGGTTCTCGATGG-3′). The amplification parameters were: 5 min denaturation at 94°C, 40 cycles of denaturation 15 s at 94°C, 15 s annealing at 60°C, and 15 s elongation at 72°C, with final extension for 10 min at 72°C. The results were normalized to an internal standard with β-actin, and gene expression was analyzed using the 2^−ΔΔCT^ method.

### Tumor Xenograft Models

BALB/c nude mice (specific pathogen-free grade, 4–5 weeks old, and 15–20 g in weight) were purchased from Guangdong Medical Laboratory Animal Center (Guangzhou, China). The animals were raised at Jinan University Experimental Animal Management Center. KYSE30-CACNA2D3 cells and KYSE30-vector cells (6 × 10^6^) were subcutaneously inoculated into the right armpit of each nude mouse. Tumor-bearing nude mice were randomly assigned to one of two groups of five mice: the treatment group mice received intraperitoneal injection cisplatin (2 mg/kg, twice per week for 4 weeks), and the control group mice were injected with PBS. Tumor volumes (mm^3^) were calculated by the formula V = 0.5 × L × W^2^. The mice were sacrificed and the tumors were isolated, weighed, and imaged. This study was carried out in accordance with the principles of the Basel Declaration and recommendations of the Guide for the Care and Use of Laboratory Animals, US National Institutes of Health (NIH Publication No. 85–23, revised 1996). The protocol was approved by the Laboratory Animal Ethics Committee of Jinan University.

### Immunohistochemistry

Immunohistochemical staining was performed with primary antibodies against CACNA2D3 (Novus Biological, Littleton, CO, USA). Sections of xenografts from mice were deparaffinized with xylene and rehydrated in alcohol baths, then incubated in 3% hydrogen peroxide for 20 min to block endogenous peroxidase activity. Antigen retrieval was performed by microwave antigen retrieval in a citric acid buffer (pH 6.0). The slides were subsequently incubated with primary antibodies at 4°C overnight and then incubated with biotinylated secondary antibodies at room temperature for 30 min, followed by incubation with horseradish peroxidase (HRP)-streptavidin for 30 min. Finally, diaminobenzidine (DAB) staining and hematoxylin counterstaining were performed. All samples were observed through a high-power light microscope.

### TUNEL Analysis

A colorimetric TUNEL apoptosis assay kit (Beyotime, Haimen, China) was used to identify apoptotic cells in the xenograft. Sections of xenograft from mice were deparaffinized, rehydrated, and incubated with proteinase K for 5 min at 37°C. Endogenous peroxidase was inactivated by treatment with 0.3% hydrogen peroxide for 20 min at room temperature. The sections were washed in PBS and incubated with a labeling buffer containing TdT at 37°C for 1 h, before being incubated with HRP-streptavidin for 30 min. Finally, the sections were incubated with DAB solution for coloration. All samples were observed through a high-power light microscope.

### RNA Sequencing (RNA-seq) and Sequencing Analysis

Equal amounts of RNA samples were used to construct strand-specific RNA libraries, following the manufacturer's standard procedures. The libraries were sequenced on a HiSeq X Ten (Illumina, San Diego, CA, USA) platform in PE150 mode. The index of the reference genome was built using Bowtie v2.2.3 (24). Differentially expressed genes (DEGs) were identified using DESeq2 (25). The thresholds for DEGs were set as false discovery rate (FDR) ≤ 0.05 and |log_2_ fold change| ≥1. Gene ontology (GO; http://www.geneontology.org) classification analysis was performed for DEGs, including molecular function, cellular component, and biological process information (26). The Kyoto Encyclopedia of Genes and Genomes (KEGG; http://www.kegg.jp) was used for systematic analysis of the signaling pathways involving the DEGs (27), and related pathways were evaluated by gene set enrichment analysis (GSEA; http://software.broadinstitute.org/gsea/) (28).

### Western Blot Assay

Western blot analysis was performed according to conventional methods. Antibodies against Akt, phosphor-Akt, PI3K, phosphor-PI3K, and GAPDH were from Cell Signaling Technology.

### Statistical Analysis

Statistical analysis was performed using GraphPad Prism 5.0 and SPSS 19.0. Data were expressed as mean ± standard deviation (S.D.). Significant differences between two independent groups were identified by student's *t*-test and expressed as ^*^p < 0.05, ^**^p < 0.01, or ^***^p < 0.001.

## Results

### Downregulation of CACNA2D3 Is Correlated With Chemoresistance in ESCC

**Figure 1:**
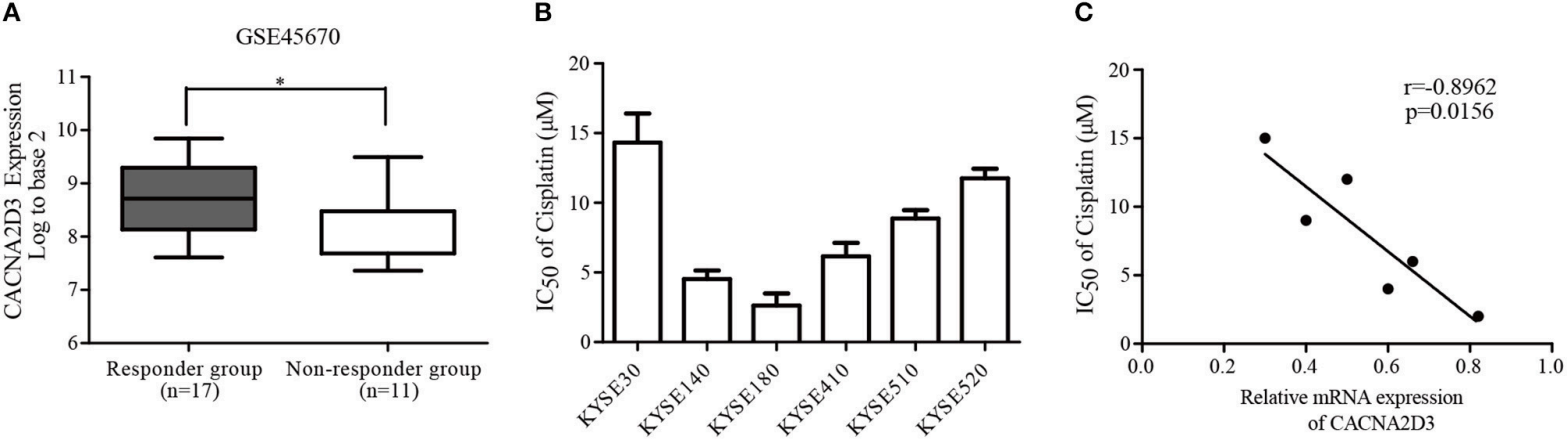
Downregulation of CACNA2D3 is associated with poor chemotherapy response in ESCC. **(A)** Box and whisker plot of CACNA2D3 mRNA levels in neoadjuvant chemotherapy responder and non-responder groups (GSE45670). ^*^p < 0.05. **(B)** IC50 values for cisplatin in six ESCC cell lines. Cells were treated with a range of concentrations of cisplatin for 72 h, and IC50 was calculated. Data are represented as the mean ± SD of three independent experiments. **(C)** Correlation of CACNA2D3 mRNA levels and IC50 values for cisplatin in six ESCC cell lines. r, Pearson correlation coefficient.

To analyze the relationship between the expression of CACNA2D3 and chemoresistance in ESCC, we screened microarray data predicting the response of esophageal cancer patients to neoadjuvant chemotherapy from the Gene Expression Omnibus (GSE45670) (29). As shown in Figure 1A, the expression of CACNA2D3 in the neoadjuvant chemotherapy responder group was significantly higher than that in the non-responder group (*p* < 0.05). We further investigated the association of CACNA2D3 expression with chemotherapeutic response in ESCC cell lines by calculating the IC50 values of cisplatin during treatment. Six ESCC cell lines were treated with different concentrations of cisplatin. The IC50 dose was calculated in each cell, and qRT-PCR was used to determine the expression level of CACNA2D3. The results showed that the IC50 value of cisplatin was negatively correlated with CACNA2D3 expression in ESCC cell lines (Figures 1B,C). These observations indicated that CACNA2D3 might regulate chemosensitivity in ESCC.

### CACNA2D3 Enhances Cisplatin Sensitivity in ESCC

**Figure 2:**
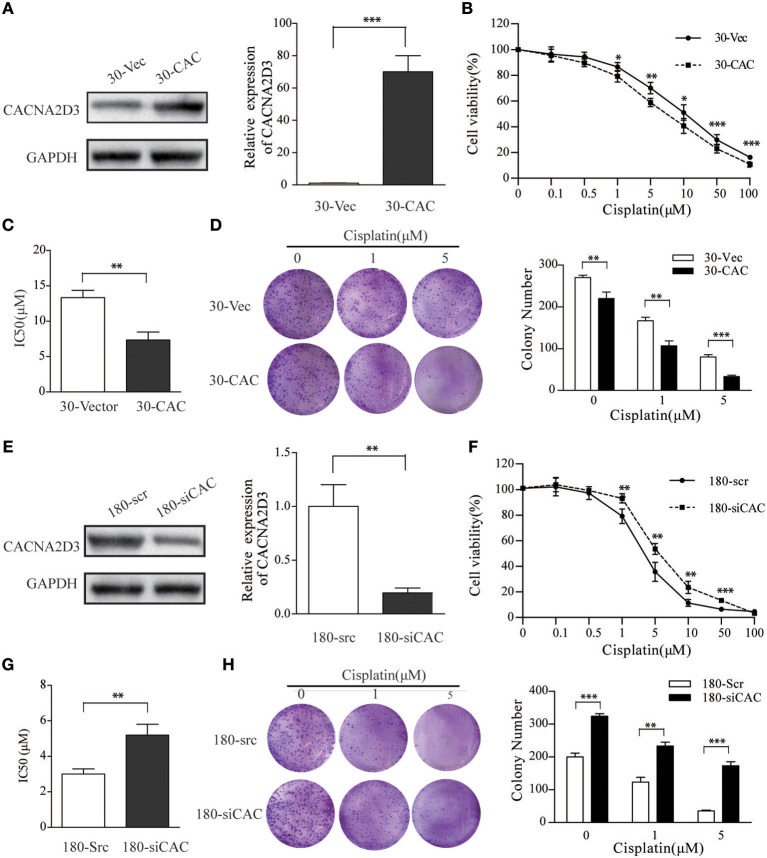
CACNA2D3 promotes chemosensitivity to cisplatin *in vitro*. **(A,E)** Stable expression of CACNA2D3 in KYSE30 cells generated by pCDNA3.1-CACNA2D3 transfection and silencing of CACNA2D3 in KYSE180 cells by siRNA were examined by western blotting and qRT-PCR. GAPDH and β-Actin were employed as a loading control. **(B,F)** CACNA2D3-overexpressing KYSE30 cells and CACNA2D3-knockdown cells were treated with cisplatin at the indicated concentrations for 72 h. The number of viable cells was measured by CCK-8 assay. **(C,G)** IC50 values were calculated using linear or logarithmic regression (*R*^2^ > 0.9). Values are presented as the mean ± SD of three wells. **(D,H)** Colony forming assays were used to determine colony forming ability after cisplatin treatment. Data are presented as the mean ± SD of three wells. ^**^p < 0.01, ^***^p < 0.001.

To investigate the role of CACNA2D3 in regulating cisplatin sensitivity in ESCC cells, a KYSE30 cell line stably expressing CACNA2D3 (30-CAC) was constructed. Control cells were transfected with empty vector (30-Vec). The expression of CACNA2D3 was determined by western blotting and qRT-PCR (Figure 2A). CCK8 assays showed that overexpression of CACNA2D3 (30-CAC) could significantly increase the cells' chemosensitivity to cisplatin compared with controls (30-Vec) (Figure 2B). The sensitivity of 30-CAC cells to cisplatin was more than twice that of 30-Vec control cells, based on the IC50 value (Figure 2C). In colony formation experiments, we also found that overexpression of CACNA2D3 combined with cisplatin could inhibit the formation of clones more significantly (Figure 2D). We next examined whether knocking down CACNA2D3 would contribute to cisplatin resistance in ESCC. The results showed that specific siRNA against CACNA2D3 could significantly suppress the expression of CACNA2D3 up to 48 h after transfection in KYSE180 (Figure 2E). Knockdown of CACNA2D3 (180-siCAC) significantly induced cisplatin resistance compared with the scrambled siRNA (180-scr) (Figure 2F). The IC50 value of 180-siCAC was higher than that of 180-scr cells (*p* < 0.001) (Figure 2G). CACNA2D3 knockdown resulted in significantly higher colony formation efficiency in 180-siCAC cells than in control cells in the presence of cisplatin (Figure 2H). Taken together, the data showed that CACNA2D3 sensitized ESCC cells to cisplatin.

### CACNA2D3 Enhances Cisplatin-Induced Apoptosis Through the Mitochondria-Dependent Pathway

**Figure 3:**
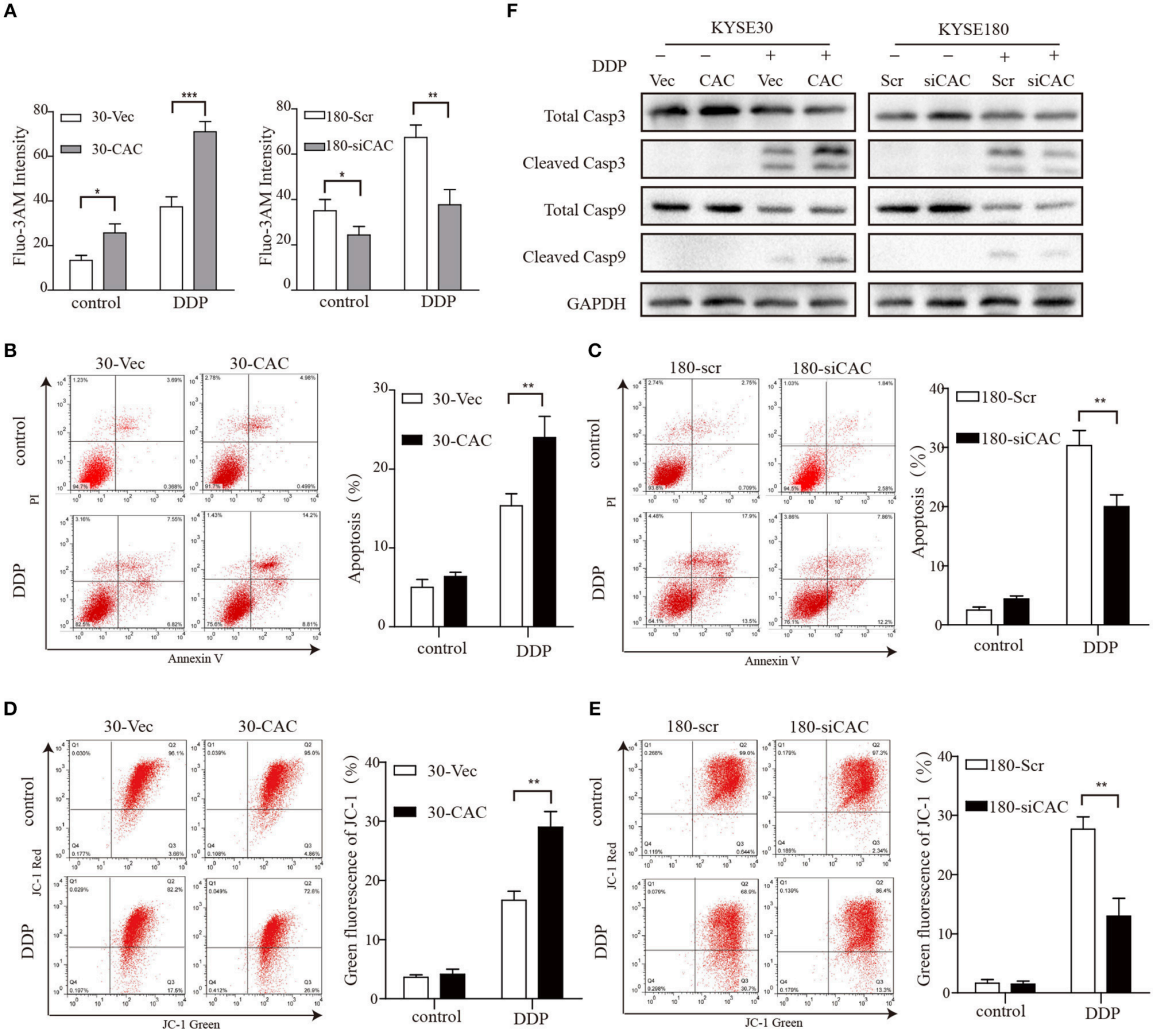
CACNA2D3 enhances cisplatin-induced apoptosis through mitochondria-dependent pathway. **(A)** Intracellular Ca^2+^ levels were detected by fluorescence-activated cell sorting (FACS) with Fluo-3 AM. **(B,C)** Annexin V/PI staining in CACNA2D3 overexpression and knockdown cell lines treated with cisplatin for 48 h; apoptosis was analyzed by FACS. The percentages of apoptotic cells are presented as the mean ± SD of three independent experiments. **(D,E)** The mitochondrial membrane potential was measured using fluorescent dye JC-1 after treatment with cisplatin for 48 h. Percentages of green fluorescence from JC-1 in cells are presented as the mean ± SD of three independent experiments. **(F)** Levels of cleaved caspase-3, total caspase-3, cleaved caspase-9, and total caspase-9 proteins were analyzed by western blotting in CACNA2D3-overexpressing and knockdown cell lines treated with or without cisplatin. ^*^p < 0.05; ^**^p < 0.01; ^***^p < 0.001.

CACNA2D3, as a regulatory subunit, has been reported to elevate the influx of extracellular Ca^2+^ into cells. In our study, Ca^2+^ levels were detected by Fluo-3 AM staining to evaluate their relationship with CACNA2D3 expression levels. CACNA2D3-overexpressing KYSE30 cells (30-CAC) showed significantly increased intracellular Ca^2+^ compared with the control cells (30-Vec), whereas knockdown of CACNA2D3 in KYSE180 cells (180-siCAC) caused a decrease in intracellular Ca^2+^ levels compared with control cells (180-scr) (Figure 3A). As Ca^2+^ can induce mitochondrial permeability changes and regulate the initiation phase of apoptosis, we performed an apoptosis assay to evaluate the effect of CACNA2D3 on the apoptosis of ESCC cells. Surprisingly, CACNA2D3 overexpression did not affect ESCC cell apoptosis, but promoted cisplatin-induced apoptosis. The percentage of apoptotic cells in 30-CAC cells increased by 24.8 ± 3.9% with cisplatin treatment, compared with 13.3 ± 1.7% in 30-Vec cells. In KYSE180 cells, cisplatin increased apoptosis by 14.2 ± 2.3% in 180-siCAC cells and by 32.3 ± 3.2% in 180-scr cells (Figures 3B,C). In addition, the JC-1 probe was used to assess changes in mitochondrial membrane potential in ESCC cells treated with cisplatin. As shown in Figures 3D,E, with cisplatin treatment, the membrane potential of 30-Vec cells decreased by 14.3 ± 2.5%, while that of 30-CAC cells decreased by 25.5 ± 1.6%. In KYSE180 cells, the membrane potential decreased by 24.2 ± 1.8% in 180-scr cells and by 9.6 ± 3.4% in 180-siCAC cells. Western blot analysis demonstrated that the ratios of cleaved Caspase9/Caspase9 and cleaved Caspase3/Caspase3 in CACNA2D3-overexpressing KYSE30 cells were higher than those in the control cells. Conversely, these ratios decreased in 180-siCAC cells treated with cisplatin compared with 180-scr cells (Figure 3F). Taken together, these results suggested that CACNA2D3 sensitized ESCC cells to cisplatin through enhancing mitochondria-mediated apoptosis.

### CACNA2D3 Increases Cisplatin Sensitivity *in vivo*

**Figure 4:**
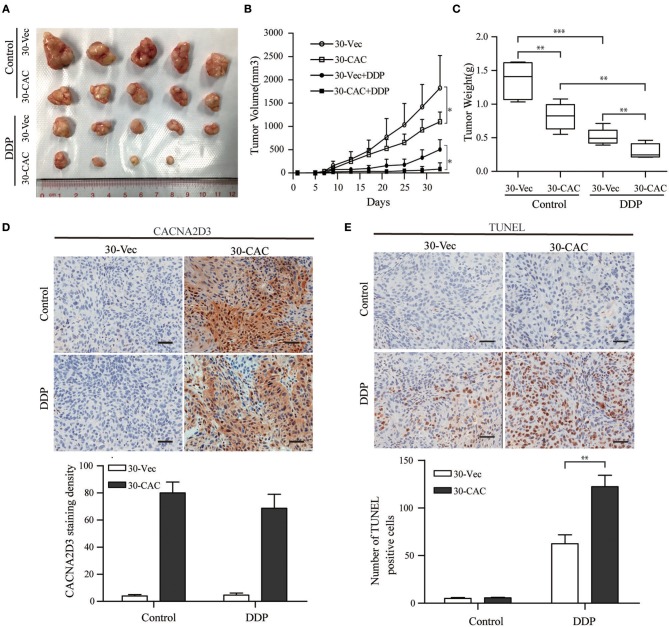
CACNA2D3 increases chemosensitivity of ESCC cells to cisplatin *in vivo*. **(A)** Representative images of xenografted tumors derived from Balb/c-nu mice at day 33. **(B)** The mice in the treatment groups were intraperitoneally injected with 2 mg/kg every 3 days. Tumor volumes were measured at 4 days intervals. Data are presented as mean ± SD. **(C)** Tumor weights were measured after mice were sacrificed. **(D)** Detection of CACNA2D3 from tumor sections by immunohistochemical staining. Quantification was performed by calculating the percentage of the staining intensities using ImageJ. **(E)** TUNEL staining of tumor sections from each group; the number of TUNEL-positive cells was quantified by counting. ^*^p < 0.05; ^**^p < 0.01; ^***^p < 0.001.

In order to better understand the role of CACNA2D3 in cisplatin sensitivity *in vivo*, we established a subcutaneous xenograft model by injecting CACNA2D3-overexpressing cells and control cells into BALB/c-nude mice. When the tumor volumes reached about 100 mm^3^, 2 mg/kg cisplatin was injected intraperitoneally twice per week for 4 weeks, while the control group received PBS. As shown in Figure 4A, we found that overexpression of CACNA2D3 and cisplatin both inhibited the growth of xenografts. However, CACNA2D3 overexpression in combination with cisplatin could more significantly inhibit the tumorigenic ability of ESCC cells. The mean tumor size (Figure 4B) and weight (Figure 4C) in the CACNA2D3 overexpression group were significantly lower than those in the vector control group after cisplatin treatment. Moreover, immunohistochemical staining showed that the expression of CACNA2D3 was increased in the CACNA2D3-overexpressing tumors compared with the control tumors (Figure 4D). TUNEL analysis also revealed that the apoptosis rate of CACNA2D3 overexpression cells was significantly higher than that of the control cells after cisplatin treatment (Figure 4E). These results together indicated that CACNA2D3 increased cisplatin sensitivity *in vivo*.

### CACANA2D3 Regulates the Sensitivity of ESCC to Cisplatin Through Inhibiting the PI3K/Akt Pathways

**Figure 5:**
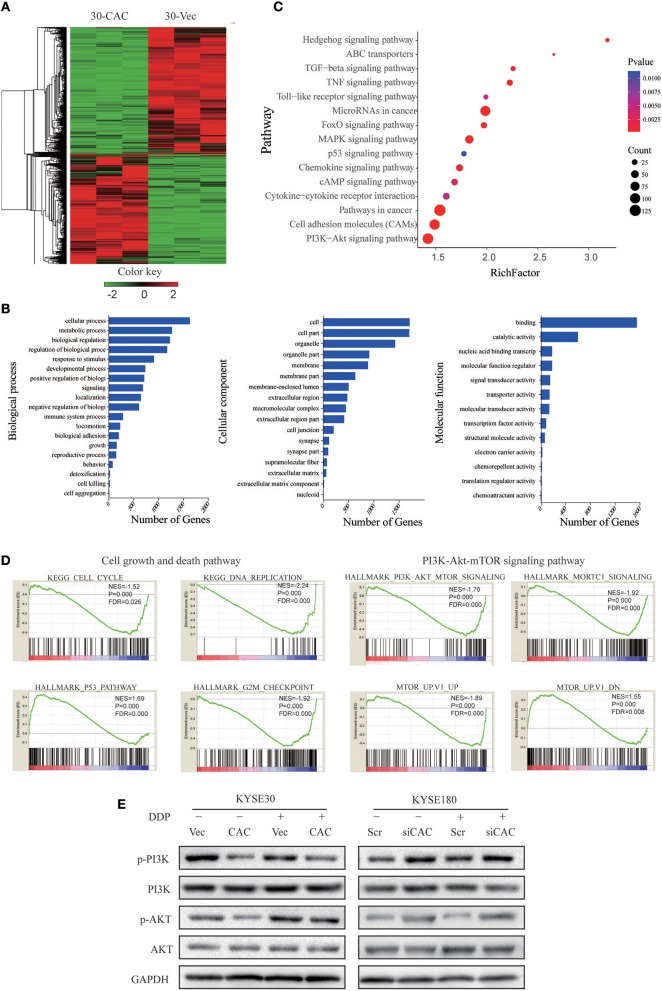
CACANA2D3 regulated the sensitivity of ESCC to cisplatin through inhibiting the PI3K/Akt pathways. **(A)** DEG heatmap and hierarchical clustering results for CACNA2D3-overexpressing KYSE30 ESCC cells. Red and green indicate high and low gene expression, respectively. **(B)** GO enrichment analysis of the DEGs. The genes were divided into three categories: cellular component, biological process, and molecular function genes. **(C)** KEGG pathway enrichment analysis of differentially expressed pathways upon CACNA2D3 overexpression. The ordinates represent the enriched KEGG pathway. *p* < 0.05 was considered statistically significant. **(D)** GSEA analysis of differentially expressed pathways upon CACNA2D3 overexpression. NES, normalized enrichment score. **(E)** Levels of P-PI3K, PI3K, P-Akt, and Akt proteins were analyzed by western blotting in CACNA2D3-overexpressing and knockdown cell lines treated with or without cisplatin.

To better understand the molecular mechanism underlying CACNA2D3-enhanced ESCC cisplatin sensitivity, we compared the gene expression profiles of CACNA2D3 stably overexpressed KYSE30 cells with those of control cells using RNA-seq after cisplatin treatment, and identified 2439 DEGs (FDR < 0.05, |log_2_ fold change| ≥1) between the two groups. A total of 1,137 genes were upregulated, and 1,302 genes were downregulated (Figure 5A). We further explored the biological functions of DEGs by GO, KEGG, and GSEA pathway enrichment analyses. Using the DAVID online GO database for comprehensive analysis, we found that CACNA2D3 was associated with multiple processes, including metabolic processes, biological regulation, regulation of biological processes, and response to stimulus (Figure 5B). KEGG database analysis revealed that multiple signaling pathway pathways were highly enriched, including “PI3K-Akt signaling pathway,” “Pathways in cancer,” “MAPK signaling pathway,” and “ABC transporters” (Figure 5C). GSEA also showed that the CACNA2D3-regulated genes were enriched in the cell growth pathway and PI3K-Akt-mTOR signaling pathway (Figure 5D). Western blotting showed that CACNA2D3 dramatically suppressed the phosphorylation of PI3K and Akt, and the suppression persisted in the presence of cisplatin (Figure 5E). These results indicated that CACNA2D3 enhanced cisplatin sensitivity by inhibiting the PI3K/Akt pathway.

### LY294002 Restores the Sensitivity of ESCC to Cisplatin in CACNA2D3-Knockdown Cells

**Figure 6:**
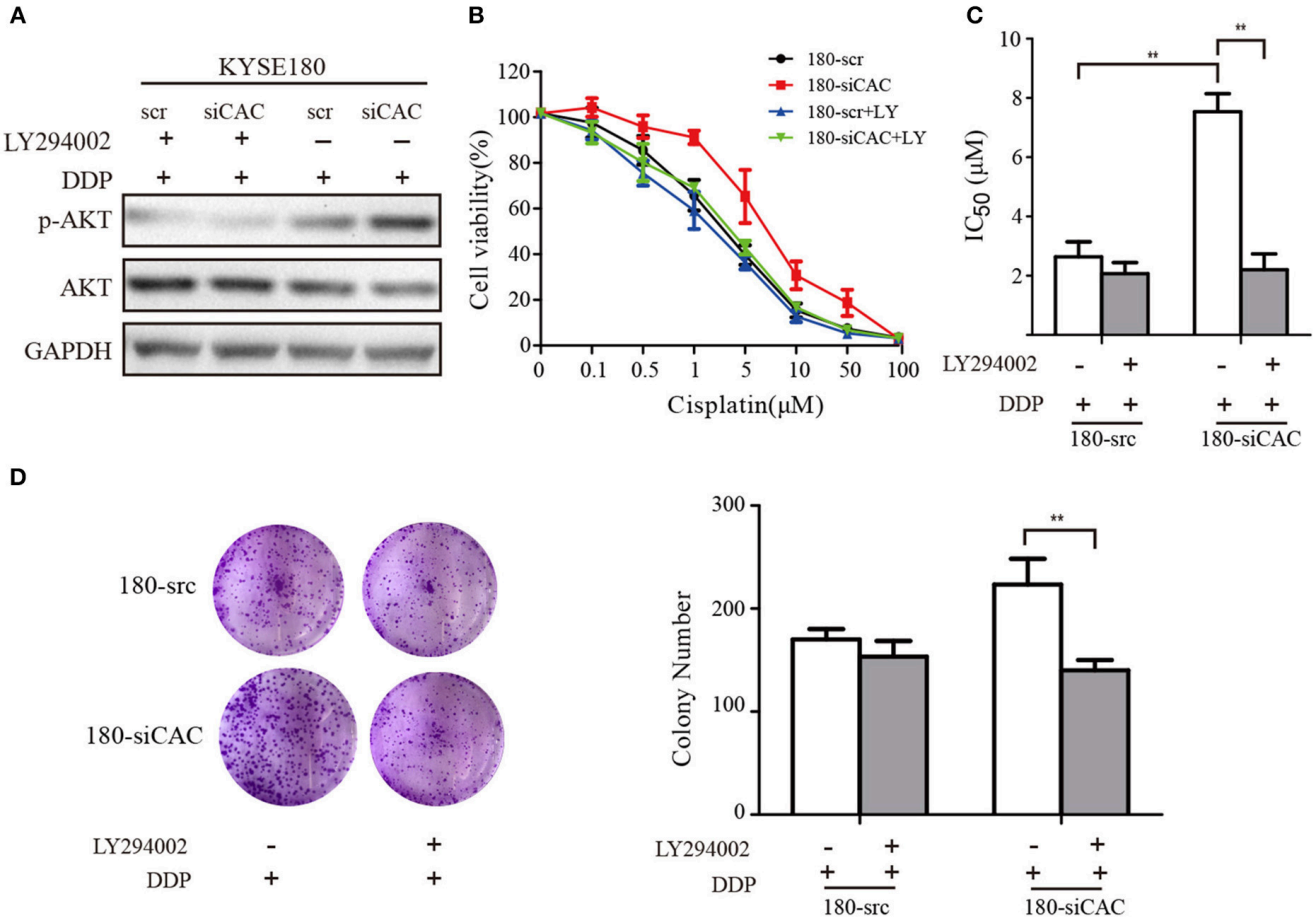
LY294002 restores the sensitivity of ESCC to cisplatin in CACNA2D3-knockdown cells. **(A)** CACNA2D3-knockdown KYSE180 cells and control cells were treated with cisplatin at the indicated concentrations with or without LY294002 for 72 h. Levels of P-Akt and Akt proteins were analyzed by western blotting. **(B)** The number of viable cells was measured by CCK-8 assay. **(C)** IC50 values were calculated using linear or logarithmic regression (R2 > 0.9). Data are presented as the mean ± SD from triplicate wells. **(D)** Colony forming assays were used to determine the colony forming ability after cisplatin with or without LY294002 treatment. Data are presented as the mean ± SD from three wells. ^**^p < 0.01.

For the rescue experiments, we treated CACNA2D3-knockdown cells with Akt inhibitor LY294002. Western blotting showed that LY294002 inhibited Akt activation in 180-siCAC cells and 180-scr cells (Figure 6A). The CACNA2D3-knockdown cells showed significantly higher sensitivity to cisplatin after treatment with LY294002 (Figure 6B). The effect of LY294002 on the IC50 reduction caused by cisplatin was significantly stronger in 180-siCAC cells than in 180-scr cells (Figure 6C). The colony formation assays also demonstrated that the combination of cisplatin and LY294002 suppressed colony formation more significantly in 180-siCAC cells than in control cells (Figure 6D). These results suggest that inhibition of the Akt signaling pathway can restore the chemosensitivity of CACNA2D3-knockdown cells to cisplatin.

## Discussion

ESCC is a cancer of the digestive system with high incidence in China. Although therapeutic strategies for ESCC have advanced considerably, its mortality rate remains high, and further efforts are needed to improve patient prognosis. Cisplatin is widely used in the clinical chemotherapy of various types of human tumors, including esophageal, gastric, testicular, bladder, ovarian, and lung cancers (30–32). However, cisplatin resistance is often the biggest obstacle to the success of chemotherapy. Therefore, it is extremely important to be able to predict cisplatin response before chemotherapy, in order to select the most appropriate treatment strategy for patients.

Calcium ions (Ca^2+^) are vital intracellular second messengers involved in multiple functions of cells, including proliferation, differentiation, fertilization, development, muscle contraction, cell death, learning, and memory (33–35). The voltage-gated calcium channel is a multi-subunit protein complex consisting of a channel-forming α1 subunit and three regulatory subunits, α2δ, β, and γ (36–38). CACNA2D3 encodes one of the α2δ subunits. Our previous study identified CACNA2D3 as a novel tumor suppressor gene for ESCC. Downregulation of CACNA2D3 predicted poor prognosis. Exogenous expression of CACNA2D3 can strongly inhibit cell growth, migration, and invasion, and induce apoptosis (21). In the current study, we first found that the expression of CACNA2D3 was higher in a platinum-based neoadjuvant chemotherapy responder group than in the non-responder group. Based on a serious of assays *in vitro* and *in vivo*, we confirmed the effect of CACNA2D3 on the chemosensitivity of cisplatin in ESCC cells.

Cisplatin is a conventional chemotherapy drug. It is activated upon entering the cell, when its chloride atoms are replaced by water molecules. The resulting hydrolytic product is an effective electrophilic reagent, which can react with any nucleophile, including DNA, RNA, and proteins. DNA is the primary target of cisplatin. Cisplatin tends to bind to the N7 site of purine bases to form a DNA adduct, causing DNA damage in cancer cells, blocking cell division, and leading to apoptosis (39–41). Several mechanisms of cisplatin resistance have been discovered, including reduced intracellular drug accumulation, increased activity of efflux pumps, changed drug targets, lost mismatch-repair ability, and escape apoptosis (42–44). The voltage-dependent calcium channel α2δ subunits have been found to regulate extracellular Ca^2+^ influx (45). Our study consistently demonstrated that the overexpression of CACNA2D3 increased the uptake of intracellular free Ca^2+^ in ESCC. Apoptosis is closely related to increased intracellular Ca^2+^ concentration. Here, we found that CACNA2D3 overexpression did not in itself affect the apoptosis rate of ESCC cells; however, it significantly increased cisplatin-induced apoptosis. Mitochondria often have a decisive role in stimulus-induced apoptosis. Mitochondrial membrane destruction and infiltration are common phenomena related to apoptosis. Excessive Ca^2+^ accumulation inside mitochondria is thought to be a powerful apoptosis stimulator that induces mitochondrial membrane depolarization and activates downstream caspases and finally induces apoptosis (46, 47). We confirmed that ectopic expression of CACNA2D3 led to depolarization of the mitochondrial membrane potential after cisplatin treatment. Moreover, the immunoblotting results showed that CACNA2D3 overexpression activated caspase-3 and caspase-9 in ESCC cells. CACNA2D3 and cisplatin synergistically induce apoptosis by increasing Ca^2+^-dependent collapse of mitochondrial membrane potential, indicating that CACNA2D3 enhances cisplatin-induced apoptosis by activating the mitochondrial pathway.

To systematically investigate the underlying molecular mechanism mediating CACNA2D3-induced ESCC cisplatin sensitivity, we compared the expression profiles of KYSE30 cells with and without CACNA2D3 overexpression after cisplatin treatment using RNA-seq. By pathway enrichment analyses, we found that CACNA2D3 could inhibit DNA replication and block ESCC cells in the G2/M phase by inhibiting the expression of p53, as shown in our previous study (21). We also found the PI3K/Akt pathway to be inactivated in CACNA2D3-overexpressing ESCC cells. The PI3K/Akt signaling pathway has important roles in promoting cell growth, proliferation, invasion, angiogenesis, and drug resistance. In-depth studies of the relationship between the PI3K/Akt signaling pathway and drug resistance have led to this pathway being considered as a new target for chemotherapy drug resistance therapy (48, 49). Here, we found that the phosphorylation of PI3K and Akt was blocked in CACNA2D3-overexpressing KYSE30 cells. Consistently, when CACNA2D3 was knocked down in KYSE180 cells, the phosphorylation level of Akt showed a significant increase. Interestingly, our data also showed that blockade of the PI3K/Akt pathway by LY294002 in CACNA2D3-knockdown cells could restore chemosensitivity to cisplatin.

In summary, in this work we first proved that the expression of CACNA2D3 was associated with chemosensitivity in ESCC patients treated with cisplatin-based therapy. Moreover, CACNA2D3 increased chemosensitivity to cisplatin in cell experiments and xenograft tumors, indicating that it could be used as a tumor marker to predict and improve patients' response to cisplatin. We further found that CACNA2D3 regulated cisplatin-induced apoptosis and decreased Akt phosphorylation. Detection of CACNA2D3 expression might be helpful for individualized treatment of ESCC patients.

## Author Contributions

CN, XC, and AH designed the study. CN, XQ, XL, YZ, YJ, YL, and QW performed the experiments. CN, XQ, XL, DZ, and BT analyzed the data. CN, XL, and XC wrote the paper.

### Conflict of Interest Statement

The authors declare that the research was conducted in the absence of any commercial or financial relationships that could be construed as a potential conflict of interest.
